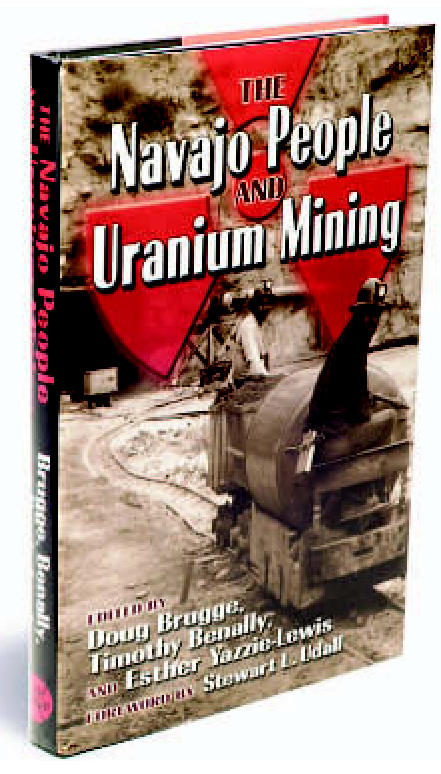# The Navajo People and Uranium Mining

**Published:** 2007-04

**Authors:** Alexandra C. Miller

**Affiliations:** *Alexandra C. Miller is a senior scientist with the Armed Forces Radiobiology Research Institute and an assistant professor at the Uniformed Services University of the Health Sciences in Bethesda, Maryland. Dr. Miller has numerous publications in the area of depleted uranium health hazards and is the author of* Depleted Uranium, Properties, Uses, and Health Consequences *from the CRC Press.*

Edited by Doug Brugge, Timothy Benally, and Esther Yazzie-Lewis

Albuquerque:University of New Mexico Press, 2006. 210 pp. ISBN: 0-8263-3778-3, $29.95

*The Navajo People and Uranium Mining*, edited by Doug Brugge, Timothy Benally, and Esther Yazzie-Lewis, is an easy and informative read. The book tells an important and heretofore untold story about the experiences of Navajo miners and families in the uranium mining industry. With a stirring foreword written by Stewart L. Udall, former U.S. Representative for Arizona and Secretary of the Interior under presidents John F. Kennedy and Lyndon Johnson, the book clearly intends both to be educational and to evoke an emotional response from the reader. The book is informative and thought provoking.

*The Navajo People and Uranium Mining* is a collection of 12 chapters. Seven chapters contain oral histories and narratives that reveal the experiences of the Navajo people from diverse perspectives, including history, psychology, culture, advocacy, and policy. These narratives, obtained through interviews, are told directly by the miners themselves or by their families, and are thus steeped in emotion. They also create a powerful documentary of the experiences of the Navajo miners, which have been largely unknown by most Americans. As with other dangerous industries in the United States, the self-sacrificing stories of the workers are usually the forgotten ones.

The other chapters in the book describe the social, cultural, and political aspects of the Navajo people and uranium mining. The authors describe the health effects of uranium and how these medical issues adversely affected the lives of the miners and their families. A lack of proper health care following uranium exposure led to further consequences. Attention is also given to the psychological consequences of uranium mining, including post-traumatic stress disorders and their harmful effects on the families. The authors also discuss the political aspects of uranium mining, including effects on the environment and the use of uranium in weapons. Furthermore, these chapters provide information on the legal aspects of uranium mining and the Navajo miners. The authors provide a detailed description of the 1990 Radiation Exposure Compensation Act and the history of its passage so that the reader can put other chapters into perspective.

Despite the excellent oral histories, the book contains some small flaws. The book is intended to evoke an emotional and empathic response from the reader and it does so in a powerful and honest way. However, the authors themselves acknowledge that many aspects of the book “are not superficially precise as quantitative science.” It is the “quantitative science” that enables medical research to help uranium workers and the medical community understand risk and the health hazards of uranium mining. In the chapters that cite studies related to uranium health effects, some references are not peer-reviewed publications but are advocacy books or transcripts of Senate hearings. Furthermore, in some chapters referencing peer-reviewed literature, the conclusions often are based more on advocacy and opinion than on the scientific facts.

*The Navajo People and Uranium Mining* is, however, an excellent book that helps to fill the information void regarding the Navajo people and their uranium mining experiences. This book provides for the Navajo people access to a large audience of readers to whom they can tell their personal and unheard stories. As a scientist who has studied uranium health hazards using objective methods, I found it nice to read a book that endeavors not only to educate but to personalize the experiences of the people our research is supposed to help.

## Figures and Tables

**Figure:**